# The Role of Endoscopic Ultrasound (EUS) in the Management of Gastric Varices

**DOI:** 10.1007/s11894-020-00801-2

**Published:** 2021-01-02

**Authors:** Sushrut Sujan Thiruvengadam, Alireza Sedarat

**Affiliations:** 1 Division of Digestive Diseases, UCLA Vatche and Tamar Manoukian, Los Angeles, CA USA; 2 Los Angeles, USA; 3 Santa Monica, USA

**Keywords:** Gastric varices, Endoscopic ultrasound, Cyanoacrylate injection, Coil injection

## Abstract

**Purpose of Review:**

Gastric varices (GV) are an important complication of portal hypertension, and the current recommendation for management is beta-blocker therapy for primary prophylaxis and transjugular intrahepatic portosystemic shunt (TIPS) for active bleeding or secondary prophylaxis. Direct endoscopic injection of cyanoacrylate (CYA) glue has been investigated but has drawbacks including limited endoscopic characterization of GV and possible distal glue embolism. To this end, endoscopic ultrasound (EUS) has been pursued to help in characterization of GV, visualization of treatment in real time, and confirmation of obliteration with Doppler.

**Recent Findings:**

In this paper, we review treatments for GV involving EUS, including EUS-guided injection of CYA and coils, either alone or in combination. We also discuss less common methods, including EUS-guided injection of thrombin and absorbable gelatin sponge. We then review literature comparing EUS-guided methods with direct endoscopic therapy and comparing individual EUS-guided methods with one another. We conclude by highlighting drawbacks of EUS in this field, including the unproven benefit over conventional therapy, lack of a standardized approach, and limited availability of expertise and necessary materials.

**Summary:**

Novel EUS-based methods offer a unique opportunity to directly visualize and access gastric varices for treatment and obliteration. This may provide key advantages over current endoscopic or angiographic treatments. Comparative studies investigating the benefit of EUS over conventional therapy are needed.

## Introduction

Among patients with portal hypertension, 20% have gastric varices (GV) [1, 2]. GV bleeding is less frequent than esophageal variceal (EV) bleeding but is more severe with higher mortality and a higher risk of rebleeding [3]. In addition to its relationship with portal hypertension, GV can also be associated with portal or splanchnic thrombosis. The Sarin classification divides GV based on its relationship with EV and its location in the stomach. GOV1 are EV extending into the lesser curvature, while GOV2 are EV extending into the fundus. IGV1 are isolated fundal varices, while IGV2 are isolated varices elsewhere in the stomach [4]. GOV1, commonly referred to as junctional varices, are the most common subtype and are managed in the same manner as EV [2]. GOV2 and IGV1 are referred to as cardiofundal varices and, compared to GOV1, are more difficult to control when they bleed with a higher risk of recurrence and a higher mortality [2]. IGV2 are uncommon, particularly in cirrhosis [1].

## Current Management of Gastric Varices

The evidence supporting current management recommendations for GV is less robust than it is for EV. In general, there is agreement that GOV1 should managed as EV [1]. However, the management of cardiofundal varices is less clear. In the 2016 AASLD guidelines, the authors separated management of GOV2/IGV1 into three categories: primary prophylaxis, acute treatment, and secondary prophylaxis of GV hemorrhage. For primary prophylaxis, the AASLD recommends non-selective beta-blockers (NSBB) [1]. For acute hemorrhage, in addition to standard medical therapy, transjugular intrahepatic portosystemic shunt (TIPS) is recommended. For secondary prophylaxis, TIPS or balloon-occluded retrograde transvenous obliteration (BRTO) is first line [1]. BRTO is a procedure that requires a gastrorenal shunt to be present and, unlike TIPS, does not divert blood flow but might increase portal pressures [5]. Therefore, some advocate selecting TIPS over BRTO if the hepatic venous portal gradient exceeds 12 mmHg [1].

## Direct Endoscopic Injection Therapy of GV

While TIPS/BRTO are recommended for GV, these methods have limitations. TIPS can be complicated by encephalopathy [6] and by shunt dysfunction causing rebleeding [7]. BRTO increases portal pressure and can worsen ascites [8]. As an alternative to TIPS/BRTO, direct endoscopic injection (DEI) of tissue adhesives into GV has been proposed. This was first described in a case report by Soehendra et al. in 1986, where the authors performed DEI with cyanoacrylate (CYA) glue in a patient with gastric varices with prior bleeding [9]. After endoscopic treatment, there was no further recurrence of bleeding [9].

Since this data was published, DEI using tissue adhesives has undergone further investigation. The two main classes of tissue adhesives are synthetic glues and biologic adhesives such as thrombin [10]. The majority of the current literature looks at direct endoscopic injection of the synthetic glue CYA (DEI-CYA) into GV. CYA is a substance that rapidly polymerizes and solidifies on contact with water or blood. The three main CYA formulations used in DEI are N-butyl-2-cyanoacrylate (NBCA), 2-octyl-cyanoacrylate (2OCA), and Glubran 2, which is NBCA plus methacryloxysulfolane [10]. Generally, NBCA has a faster polymerization time than 2OCA or Glubran-2 (Table 1) [10, 11]. Therefore, in order to prevent premature solidification and adherence to equipment, NBCA is often mixed with lipiodol, an oily contrast media that slows polymerization time [10, 11].

**Table 1:** Polymerization times of different cyanoacrylate formulations

CYA type	Oil used	Polymerization	
		Speed	Time (s)
NBCA	N	Faster	4–5
	Y	Slower	10–19
2-OCA	N	Slower	7.5–16
	Y	Slower	10–54
Glubran-2	N	Slower	

Ex-vivo polymerization times are measured with a slide test in which a drop of CYA is added to a drop of blood on a microscope slide. Results with or without mixture with oily contrast media (lipiodol or ethiodol) are shown. The slide test was not performed for Glubran-2, though this is recognized as having a slow polymerization time. *CYA* (cyanoacrylate), *NBCA* (N-butyl-2-cyanoacrylate), *2-OCA* (2-octyl-cyanoacrylate), *Glubran-2* (NBCA plus methacryloxysulfolane). Adapted from Caldwell [11] and the ASGE technology status evaluation report on tissue adhesives [10]

While DEI-CYA is relatively safe, its most feared complication is distal embolization caused by downstream flow of glue before polymerization. Pulmonary embolism is believed to occur in up to 5% of DEI-CYA cases and other embolic events, including splenic infarction, portal vein embolization, septic emboli, stroke, and coronary emboli have been reported [3]. Other adverse events of DEI-CYA include transient abdominal pain and fever [12]. Instrument damage due to polymerization of CYA in the working channel and adherence to the scope tip has also been reported [13].

At this time, literature comparing DEI-CYA to conventional GV treatment is limited. For primary prophylaxis, Mishra et al. compared DEI-CYA to NSBB or no treatment and found that DEI-CYA decreases probability of GV bleeding [14]. For acute bleeding, Castellanos et al. performed a meta-analysis comparing DEI-CYA to variceal ligation and found no difference with regard to control of bleeding or mortality, but did note that DEI-CYA had a lower risk of rebleeding [15]. Procacinni et al. performed a retrospective analysis comparing DEI-CYA with TIPS for acute GV hemorrhage and found no difference in rebleeding, although the TIPS group had higher rates of morbidity requiring hospitalization [16]. For secondary prophylaxis, Lo et al. compared DEI-CYA to TIPS and found that DEI-CYA had higher rebleeding rates with greater transfusion requirement [17].

Given this limited data, endoscopic therapy has not been formally recommended for GV. The AASLD guidelines state that DEI-CYA is an “option for cases in which TIPS is not feasible […] in centers where the expertise is available” [1]. Similarly, the Baveno VI consensus notes that “further studies are needed to evaluate the risk/benefit ratio of using cyanoacrylate” [18]. EASL guidelines note that “cyanoacrylate is the recommended endoscopic hemostatic treatment for cardiofundal varices” but does not comment on whether DEI-CYA should be pursued over TIPS/BRTO [2].

## The Diagnostic Role of Endoscopic Ultrasound in GV Management

While direct endoscopic injection with CYA has promise, there are several limitations. Importantly, during a standard upper endoscopy, the presence of gastric varices is often difficult to determine, particularly with active bleeding [19]. Furthermore, estimates of size and presence of feeding vessels are not always easily determined. This is particularly important as risk factors for GV rebleeding include varix size and the presence of para-gastric veins [20]. For this reason, endoscopic ultrasound (EUS) may have benefits in GV management, as it can better visualize the gastric wall and associated vasculature even in the setting of an active bleed [21].

Another potential drawback of DEI-CYA is that confirmation of variceal obliteration by standard endoscopy is subjective and relies on determining “hardening” of the varix post-injection [22•]. This is particularly important as the risk of potentially fatal embolization increases with the amount of CYA injected [23]. Having a more precise manner of determining obliteration can minimize the amount of glue injected.

For this reason, in 2000, Lee et al. examined the role of EUS monitoring to determine GV obliteration. They performed a prospective study comparing patients undergoing DEI-CYA for acute GV hemorrhage who did or did not undergo regular EUS surveillance to verify obliteration. If EUS showed flow within the varix, repeat DEI-CYA was performed. The authors found that EUS monitoring led to total obliteration in 80% of cases and significantly reduced rates of recurrent bleeding compared to DEI-CYA alone [24]. Mosli et al. found that patients who underwent DEI-CYA followed by post-eradication EUS appeared less likely to have recurrent GV compared to patients who underwent DEI-CYA alone (2 of 11 patients vs 5 of 11 patients), though the number of patients was low and no statistical comparison was performed [25].

## The Therapeutic Role of Endoscopic Ultrasound in GV Management

While EUS may be useful as a diagnostic adjunct, its therapeutic potential has gained greater recognition over the past few years. Under EUS guidance, different hemostatic adhesives and devices can be injected into GV including CYA (EUS-CYA), coils (EUS-coil), coils with CYA (EUS-coil/CYA), thrombin (EUS-thrombin), and coils with absorbable gelatin sponge (EUS-coil/AGS). Data on safety and efficacy of these methods is summarized in Table 2.

**Table 2:** Safety and efficacy of EUS-guided treatments for gastric varices

Technique	Study	Year	Sample Size	Follow-up (in months)	Assessment of GV obliteration	GV obliteration Rate	Rebleeding Rate	AE Rate	Severe AE Rate	Severe AEs	Notes
EUS-CYA	Romero-Castro et al	2007	5	10	Index EUS	5 (100%)	0 (0%)	0 (0%)	0 (0%)		
	Gonzalez et al	2012	3	9	Index EUS	3 (100%)	0 (0%)	0 (0%)	0 (0%)		
	Romero-Castro et al	2013	19	21	Index EUS	19 (100%)	0 (0%)	11 (58%)	0 (0%)		9 asymptomatic PEs detected on routine CT scan performed after procedure
	Franco et al	2014	20	31	Index EUS	20 (100%)	1 (5%)	3 (15%)	0 (0%)		
	Bang et al	2015	31	8	Index EUS	31 (100%)	7 (23%)	18 (47%)	4 (8%)	PE, splenic infarct	
	Bick et al	2019	64	7	Index EUS	49 (77%)	5 (8%)	13 (20%)	3 (5%)	PE, splenic infarct	
EUS-coil	Romero-Castro et al	2010	4	5	Index EUS	3 (75%)	0 (0%)	0 (0%)	0 (0%)		
	Romero-Castro et al	2013	11	11	Index EUS	10 (91%)	0 (0%)	1 (9%)			
	Fujii-Lau et al	2016	2	8	Index EUS	2 (100%)	0 (0%)	N/A			
	Khoury et al	2019	10	10	Index EUS	7 (70%)	0 (0%)	6 (60%)	1 (10%)	Major bleeding	Of 10 patients, 6 had EUS-coil and 4 had EUS-coil/CYA
EUS-coil/CYA	Binmoeller et al	2011	30	6	Follow-up EUS	23/24 (96%)	1 (3%)	2 (7%)	0 (0%)		24 of 30 patients had follow-up EUS
	Bhat et al	2016	152	15	Follow-up EUS	93/100 (93%)	20/125 (16%)	9/125 (7%)	1/125 (0.8%)	PE	125 of 152 patients had clinical follow-up, 100 of 152 patients had follow-up EUS
	Fujii-Lau et al	2016	3	4	Index EUS	3 (100%)	0 (0%)	N/A			
	Mukkada et al	2018	30	6	Follow-up EUS	8/20 (40%)	6 (20%)	N/A			15 patients underwent EUS-coil, 15 patients underwent EUS-coil/CYA. 20 of 30 patients had follow-up EUS
	Lobo et al	2019	16	10	Follow-up EUS	15/15 (100%)	0 (0%)	9 (56%)	4 (25%)	PE	15 of 16 patients had follow-up EUS
	Koziel et al	2019	16	11	Index EUS	15 (94%)	0 (0%)	6 (38%)	0 (0%)		
EUS-thrombin	Frost and Hebbar	2018	3	3	Index EUS	2 (67%)	1 (33%)	0 (0%)	0 (0%)		3 of 8 patients had active GV hemorrhage and obliteration determined on index EUS
	Frost and Hebbar	2018	5	24	Follow-up EUS	4 (80%)	0 (0%)	0 (0%)	0 (0%)		5 of 8 patients referred for prophylaxis and obliteration determined on follow-up EUS
EUS-coil/AGS	Bazarbashi et al	2020	10	7	Follow-up EUS	10 (100%)	0 (0%)	3 (30%)	0 (0%)		

For each study, rates of GV obliteration, rebleeding, adverse events and severe adverse events are shown. Some authors determined GV obliteration on the index EUS while others determined GV obliteration on follow-up EUS. *EUS* (endoscopic ultrasound), *GV* (gastric varices), *AE* (adverse events)

### EUS-CYA

EUS-guided injection of CYA was first described by Romero-Castro et al. in 2007. In this case series of 5 patients, the authors performed EUS-guided injection of a 1:1 mixture of NBCA or Glubran-2 with lipiodol into a gastric varix at the entrance of the perforating vein. In all patients, EUS-CYA was successful, and there was no recurrent bleeding or complications during the mean 10-month follow-up [26]. Since this study, others have described this technique. There is no consensus on the exact method of performing EUS-CYA. In general, a linear array echoendoscope is used [27, 28, 29•, 30, 31•], and the target for injection is the varix itself [28, 29•, 31•] though Romero-Castro et al. targeted the feeding vessel [27]. Either a 19 g [28, 30, 31•] or 22 g [27, 29•, 31•] needle is used, and CYA is injected with EUS color Doppler and fluoroscopy to allow real-time visualization of injection and GV obliteration.

EUS-CYA has been described for primary prophylaxis, acute GV hemorrhage, and secondary prophylaxis of GV. As above, Romero-Castro et al. described 5 patients who underwent EUS-CYA for primary prophylaxis with successful GV obliteration [26]. Franco et al. looked at 20 patients who underwent EUS-CYA for primary prophylaxis, and obliteration was successful in all patients. Only 1 of 20 patients had recurrent GV and recurrent bleeding [32]. For treatment of acute GV hemorrhage, Gonzalez et al. described 3 patients who underwent EUS-CYA with successful hemostasis [28]. In 2013, Romero-Castro et al. described a cohort of 10 patients with active GV hemorrhage who underwent EUS-CYA with successful hemostasis in all cases [27]. Gubler and Bauerfeind described 40 patients who underwent EUS-CYA for either acute bleeding or primary/secondary prophylaxis. All acute bleeds stopped, but 3 patients in the total cohort required rescue therapy with TIPS or liver transplant [29•].

EUS-CYA has a similar adverse effect profile as DEI-CYA. Abdominal pain was reported in 8–15% [31•, 32], fever in 8–9% [27, 31•], and transient bacteremia in 2–6% [29•, 31•] of patients undergoing EUS-CYA. Ulcer at the injection site was seen in 3% of patients undergoing this procedure [29•]. Systemic embolization is also seen even with EUS guidance. Romero-Castro et al. noted pulmonary embolism in 9 of 19 patients (47%) who underwent EUS-CYA; these were all asymptomatic and were detected on routine imaging performed as part of the study protocol [27]. In other studies, pulmonary embolism was noted in 2–6% of patients [31•, 33] and splenic infarcts in 2–6% of patients [31•, 33].

### EUS-Coil and EUS-Coil/CYA

As with DEI-CYA, EUS-CYA has a risk of distal embolization. For this reason, EUS-guided coil injection has been pursued either with or without CYA injection, with the hope that the coil can provide primary hemostasis and serve as a scaffold to retain glue within the varix and thus reduce the risk of embolization [34].

Coil injection in GV was first described by Romero-Castro et al. in 2010 [35]. As with EUS-CYA, there is no standardized approach to this procedure. In general, a standard linear array echoendoscope is used [19, 21, 22•, 36, 37, 38•, 39, 40], though some use a forward-viewing linear array echoendoscope [34, 41••]. The echoendoscope is either positioned in the distal esophagus for a transesophageal-transcrural approach [21, 34, 37, 41••, 42] or in the stomach for a transgastric approach [39, 40] for coil injection. Either a 19 g [19, 21, 22•, 34, 37, 38•, 39, 40, 41••, 42] or 22 g [36, 41••] needle is used, and either the varix or a feeding vessel is targeted. Coils anywhere from 5 to 20 mm in diameter [19, 22•, 34, 36, 37, 38•, 39, 40, 41••, 42] are delivered into the varix through a FNA needle using the stylet as a pusher. If glue injection is desired, CYA is immediately injected after coil deployment using the same needle [34]. Color Doppler is performed to confirm the absence of flow within the varix.

There are limited data examining coil injection alone. Romero-Castro et al. performed primary prophylaxis of GV with EUS-coil in 4 patients and found that coil placement eradicated varices in 3 patients with no complications or migrations [35]. Khoury et al. looked at 10 patients with GV who underwent EUS-coil for either primary prophylaxis or active bleeding [39]. In 7 patients, there was near complete eradication, while the remaining 3 required repeated coil injections [39]. Fujii-Lau described 2 patients who underwent EUS-coil, with GV obliteration noted in both [36].

Rather than only injecting coils, several groups advocate combining coil and glue injection, with the hopes that these two modalities act synergistically to stop bleeding while minimizing distal embolization. Bhat et al. published a large case series of 151 patients with GV who underwent successful EUS-coil/CYA, of whom 125 had clinical or endoscopic/EUS follow-up. Twenty patients (16%) had either early (*n* = 12) or late (*n* = 8) post-treatment bleeding. Of 100 patients with follow-up EUS, 73 had complete obliteration in a single procedure, 14 required additional procedures for successful obliteration, 3 could not be obliterated, and 4 were obliterated initially with residual varices detected on follow-up [41••]. This large study indicated that EUS-coil/CYA is effective in the treatment of GV.

The specific role of EUS-coil/CYA in primary prophylaxis, acute hemostasis, and secondary prophylaxis is less clear, as most studies investigating individual roles for EUS are small. Of the cohort described by Bhat et al., 27 of 28 patients who underwent EUS-coil/CYA for primary prophylaxis had complete obliteration on follow-up EUS, while the remaining patient required retreatment [41••]. Similarly, Koziel et al. performed EUS-coil/CYA for primary prophylaxis in 6 patients with documented obliteration in all patients [40]. For treatment of actively bleeding GV, Bhat et al. performed EUS-coil/CYA on 7 patients with successful hemostasis in all patients [41••]. Wang et al. described 1 patient [21], and Binmoeller et al. described 2 patients [34] with active bleeding successfully treated by this technique. In their cohort, Binmoeller et al. also found that of 24 patients referred for secondary prophylaxis, 23 had complete GV obliteration after 1 treatment with EUS-coil/CYA. Recurrent bleeding was seen in 1 patient with successful repeat treatment [34]. Similarly, Koziel et al. described EUS-coil/CYA for secondary prophylaxis in 10 patients with complete obliteration in all patients [40]. Fujii-Lau et al. also described EUS-coil/CYA for secondary prophylaxis in 3 patients, with no rebleeding episodes documented [36].

Coil placement with or without CYA can have associated adverse effects. Romero-Castro et al. observed extrusion of coils into the gastric lumen with mucosal scarring in 1 of 11 patients (9%) [27]. Minor GI bleeds from the puncture site have been reported in 6–50% of cases [22•, 39, 40], and minor bleeding from coil or CYA extrusion has been reported in 3% of cases [41••]. Major bleeding has also been observed; Khoury et al. found that 1 of 10 patients (10%) who underwent EUS-coil had major bleeding from the puncture site [39]. Coil migration is also a rare adverse event that can occur [36]. In cases where CYA is also administered, abdominal pain has been reported in anywhere from 3 to 43% of patients [22•, 40, 41••, 43], and fever has been reported in up to 13% of patients [40]. Finally, as with EUS-CYA, distal embolism remains a concern with EUS-coil/CYA; Bhat et al. observed pulmonary embolism in 1 of 125 patients (0.8%) [41••]; and Lobo et al. observed this in 4 of 16 patients (25%) [22•].

### Other Therapeutic EUS Modalities for GV

In addition to synthetic tissue adhesives such as CYA, biologic tissue adhesives have been studied for GV obliteration. Chief among these is thrombin, which converts fibrinogen to fibrin and promotes clot production, leading to hemostasis [3]. A 5 mL solution of thrombin, with a concentration of 1000 international units (IU)/mL, can clot a liter of blood in less than 60 s [3]. In a randomized controlled trial performed by Lo et al., direct endoscopic injection of thrombin (*n* = 33) was compared to DEI-CYA (*n* = 35) in the treatment of actively bleeding GV. The authors found similar rates of hemostasis at 48 h in both groups (94% vs 97%; *P* = 0.60) but found that thrombin injection was associated with a lower rate of gastric ulceration (0% vs 37%; *P* < 0.001) and with fewer complications in general (12% vs 51%; *P* < 0.001) [44]. While neither group underwent EUS-guided injection, this study indicates that thrombin may have a more favorable adverse event profile than CYA. EUS-guided injection of thrombin was first described by Frost and Hebbar. In 8 patients with GV, EUS-thrombin was performed until either there was no observable flow or until 10,000 IU was injected. Five of 8 patients underwent EUS-thrombin for primary prophylaxis with obliteration observed in 4 patients, and no future bleeding observed in any patient. In 3 patients with active bleeding, EUS-thrombin resulted in successful hemostasis in 2 patients. Unfortunately, the other patient had no alteration in blood flow. This procedure was safe in this cohort with no procedure-related complications [45].

Another synthetic tissue adhesive with potential in treating GV is absorbable gelatin sponge (AGS). AGS is prepared from purified porcine gelatin and can absorb up to 45 times its weight in whole blood. EUS-guided coil placement followed by AGS injection has positive results in small case series [42, 43]. Ge et al. described a case of 1 patient who presented with active bleeding from GV and underwent EUS-coil/AGS with trans-esophageal transcrural placement of 8 coils. AGS was then prepared into a liquid slurry, and 5 cc was injected to enhance eradication. This patient tolerated this procedure well with successful hemostasis and with obliteration persisting on follow-up EUS [42]. Bazarbashi et al. described 10 patients who underwent EUS-coil/AGS for active bleeding or secondary prophylaxis and noted lack of any rebleeding episodes from GV after a mean follow-up of 6 months. Nine of 10 patients had follow-up EUS with near complete GV obliteration in all cases. This procedure was generally well tolerated, with abdominal pain reported in 1 of 10 patients and transient low-grade fever reported in 2 of 10 patients [43].

## Comparison of Different Endoscopic Methods for GV

Much of the published literature on EUS-guided treatments for GV involves small case series. Direct comparisons between different endoscopic and EUS methods are sparse and limited in sample size. Therefore, meta-analyses comparing different methods have been performed.

### Comparisons Between Direct and EUS-Guided Endoscopic Methods

Studies comparing DEI-CYA and EUS-CYA suggest benefit with EUS guidance. Bang et al. performed a prospective study between 2006 and 2015, where 40 patients prior to 2012 received DEI-CYA for GV treatment and 31 patients after this point received EUS-CYA. While EUS-CYA had a lower incidence of GV rebleeding (8 vs 24%; *P* = 0.176), this was not statistically significant. Recurrent all-cause GI bleeding was significantly lower in the EUS-CYA group (23% vs 58%; *P* = 0.004). Adverse events were more likely in the EUS-CYA group (47% vs 13%; *p* < 0.001), but incidence of moderate/severe adverse events did not differ between the two groups [33]. Bick et al. also compared these two techniques in a retrospective analysis of 64 patients who underwent EUS-CYA and 40 patients who underwent DEI-CYA for GV treatment. Patients in the EUS-CYA group had lower mean volume of CYA injected (2.0 vs 3.3 mL; *P* < 0.001) with greater number of varices injected (1.6 vs 1.1 varies; *P* < 0.001). Furthermore, GV rebleeding was less frequent in the EUS-CYA group (9% vs 24%; *P* = 0.045) with a similar adverse event profile (20% vs 18%; *P* = 0.723) [31•].

Other EUS-guided methods also appear to have benefit over DEI-CYA. Mukkada et al. performed a retrospective analysis comparing 30 patients who underwent EUS-guided coil injection (EUS-coil in 15 patients and EUS-coil/CYA in 15 patients) with 51 patients who underwent DEI-CYA for secondary prophylaxis of GV bleeding. Patients in the EUS group had a significantly lower rate of rebleeding compared to the DEI-CYA group [38•]. In another study, Lobo et al. performed a prospective randomized controlled trial in which 32 patients were randomized to either EUS-coil/CYA (16 patients) or DEI-CYA with EUS to verify eradication (16 patients) for primary or secondary prophylaxis. There was no significant difference with regard to GV obliteration. However, a smaller amount of CYA was needed for obliteration in the EUS-coil/CYA group (1.40 vs 3.07 mL; *P* = 0.002). Asymptomatic PE occurred in 25% of EUS-coil/CYA group versus 50% of DEI-CYA, which trended toward significance (*P* = 0.144) [22•].

While these data suggest benefit to EUS-guided therapy over conventional endoscopic injection, much of the published literature involves small numbers of patients. Therefore, Mohan et al. recently performed a meta-analysis comparing EUS-guided treatment of GV with DEI-CYA using data from 23 published studies. There was no difference in pooled treatment efficacy between EUS-guided therapy and DEI-CYA (94% vs 91%; *P* = 0.4), but the pooled rate of GV obliteration was significantly higher in the EUS group (84% vs 63%; *P* = 0.02). There was no difference in early rebleeding (7% vs 5%; *P* = 0.7), but the EUS group had a lower rate of late rebleeding (12% vs 17%; *P* = 0.1) and GV recurrence (9% vs 18%; *P* = 0.06) in differences which trended toward statistical significance [46••]. This meta-analysis suggests that EUS-guided therapy is superior to direct endoscopic injection. Future large, randomized controlled trials will be needed to verify these findings.

### Comparison Between Different EUS-Guided Methods

Direct comparisons between EUS-guided methods are sparse. Romero-Castro et al. compared coil with CYA injection in a retrospective analysis of 11 patients who underwent EUS-coil and 19 patients who underwent EUS-CYA. There was no difference in GV obliteration rate between EUS-coil and EUS-CYA (91% vs 100%). However, patients who underwent EUS-coil had fewer adverse events (9% vs 58%; *P* < 0.1) though symptomatic adverse events did not differ between the two groups (9% vs 11%; *P* > 0.5) [27]. This small study indicates that both EUS-coil and EUS-CYA are effective and do not appear to show a distinct advantage of one method over the other.

As noted above, combining coil and CYA injection has a theoretical benefit over CYA injection alone as coils act as a scaffold for CYA which may enhance obliteration while decreasing likelihood of embolic spread of CYA [34]. In the meta-analysis by Mohan et al., the authors found that EUS-coil/CYA had significantly fewer instances of GV recurrence than EUS-CYA (5.2% vs 15%; *P* = 0.01) [46••]. McCarty et al. performed a meta-analysis of 11 studies comparing EUS-guided methods and found similar advantages to the combined approach. Their results showed that EUS-coil/CYA had a significantly higher rate of GV obliteration than either EUS-CYA (98% vs 96%; *P* < 0.001) or EUS-coil (98% vs 90%; *P* < 0.001) and had a lower need for reintervention than either EUS-coil (15% vs 26%; *P* < 0.001) or EUS-CYA (15% vs 25%; *P* = 0.047). EUS-coil/CYA also had a lower rebleeding rate than EUS-CYA (14% vs 30%; *P* < 0.001) but did not differ from EUS-coil in this regard (14% vs 17%; *P* = 1). The frequency of adverse events was also lower in the EUS-coil/CYA group compared to EUS-CYA (10% vs 21%; *P* < 0.001) but did not differ when compared to EUS-coil [47••].

Together, this data from individual studies and meta-analyses indicate that EUS-guided coil and CYA injection have the best efficacy in the treatment of GV. Of note, the majority of these studies was retrospective and non-randomized prospective studies. The randomized controlled trials included in meta-analyses were small, with anywhere from 16 to 30 patients enrolled [46••, 47••]. Therefore, additional large, well-designed randomized-controlled trials are needed to confirm the potential benefit of EUS-coil/CYA on the treatment of GV.

## Conclusion

Despite the promise of EUS in the treatment of GV, the benefit of EUS-guided treatment over conventional therapy has not yet been demonstrated. To date, there are no head-to-head studies comparing EUS-guided treatments to NSBB therapy for primary prophylaxis or TIPS/BRTO for active bleeding or secondary prophylaxis. Bhat et al. proposed an algorithm for treating cardiofundal varices with EUS [41••]. For primary prophylaxis, the authors recommend EUS [41••]. If this shows a dominant varix 20 mm or greater in size, EUS-coil/CYA should be pursued [41••]. For bleeding due to IGV1/GOV2, EUS-coil/CYA should be pursued for hemostasis and obliteration [41••]. If hemostasis is not achieved, salvage therapy, including TIPS, is recommended [41••]. Future studies are needed to determine whether this EUS-based approach has benefit over the current guideline recommendations for GV treatment.

The role of EUS in the management of GV has certain limitations that merit further discussion. As with direct endoscopic injection, EUS-guided treatments require significant expertise and may not be readily available in all centers. Furthermore, the exact method of EUS-guided variceal injection is not standardized. Different groups use different types of echoendoscopes (side-viewing vs forward-viewing), different positions during injection (transesophageal vs transgastric), and different needle sizes. Furthermore, the exact type of CYA used varies in the literature. Another important limitation is the institutional and regional availability of necessary materials for endoscopic injection, specifically tissue adhesives such as cyanoacrylate. The FDA considers tissue adhesives “transitional devices” which are class III devices that require premarket approval [48]. This may limit the widespread adoption of endoscopic methods involving CYA in the USA. Future efforts should continue to focus on demonstrating safety and efficacy of EUS-guided injection of tissue adhesives in order to obtain FDA approval.

In conclusion, EUS-guided treatment of gastric varices is a novel technique which has the potential to significantly improve our management of this condition. EUS guidance allows for clear visualization and precise targeting of gastric varices and feeding vessels even in the setting of active bleeding, which is not always possible during conventional endoscopy. Furthermore, this superior imaging of varices decreases the amount of therapeutic agents needed to achieve obliteration, which may in turn reduce associated adverse effects. Furthermore, unlike angiographic methods such as BRTO or TIPS, EUS-guided injection of specific vessels is not associated with significant increases in portal hypertension leading to ascites or shunting leading to hepatic encephalopathy. While these potential advantages of EUS-guided treatment of GV are promising, future studies are needed to demonstrate superiority over conventional medical and radiologic therapies. Additional efforts to standardize EUS-guided methods and obtain approval for its regular use in the USA will also be crucial.

## Abbreviations

BRTO: Balloon-occluded retrograde transvenous obliteration
CYA: Cyanoacrylate
DEI: Direct endoscopic injection
DEI-CYA: Direct endoscopic injection of cyanoacrylate
EUS: Endoscopic ultrasound
EUS-CYA: Endoscopic ultrasound-guided injection of cyanoacrylate
EUS-coil: Endoscopic ultrasound-guided injection of coils
EUS-coil/CYA: Endoscopic ultrasound-guided injection of coils with cyanoacrylate
EUS-coil/AGS: Endoscopic ultrasound-guided injection of coils with absorbable gelatin sponge
EUS-thrombin: Endoscopic ultrasound-guided injection of thrombin
EV: Esophageal varices
GV: Gastric varices
IU: International units
NBCA: N-Butyl-2-cyanoacrylate
2OCA: 2-Octyl-cyanoacrylate
TIPS: Transjugular intrahepatic portosystemic shunt
